# The genus *Camptochaeta* in Nearctic caves, with the description of *C. prolixa* sp. n. (Diptera, Sciaridae)

**DOI:** 10.3897/zookeys.135.1624

**Published:** 2011-10-07

**Authors:** Pekka Vilkamaa, Heikki Hippa, Steven J. Taylor

**Affiliations:** 1 Zoological Museum, Finnish Museum of Natural History, P.O. Box 17, FI-00014 University of Helsinki, Finland; 2 Swedish Museum of Natural History, P.O. Box 50007, SE-104 05 Stockholm, Sweden; 3 Illinois Natural History Survey, University of Illinois at Urbana-Champaign, 1816 S Oak St, Champaign, Illinois USA

**Keywords:** Diptera, Sciaridae, *Camptochaeta*, new species, new records, caves, USA

## Abstract

*Camptochaeta prolixa* **sp. n.** (Diptera, Sciaridae) is described from caves in Nevada, and three other congeneric species are recorded from caves in Nevada and Arkansas, United States. The new species shows some indication to a subterranean mode of life, including long antenna and legs, and in some specimens, reduction of the eye bridge.

## Introduction

The genus *Camptochaeta* Hippa & Vilkamaa, 1994 includes three species which have been found in caves: *Camptochaeta ofenkaulis* (Lengersdorf, 1925) and *Camptochaeta scanica* Hippa & Vilkamaa, 1994 and *Camptochaeta subcamptochaeta* (Mohrig, 1992) (see Mohrig and Eckert 1992). Of these, *Camptochaeta scanica *has been found also outside caves.

Regarding the Nearctic Sciaridae, only specimens of ‘*Corynoptera* sp.' have been recorded from a cave in Arkansas (Graening et al. 2003).

Between May 2006 and July 2007, a bioinventory of caves at Great Basin National Park, White Pine County, Nevada was undertaken, and that study has already led to the description of several new species, including two millipeds (Shear 2007, Shear et al. 2009), a springtail (Zeppilini et al. 2009) and an amphipod (Taylor and Holsinger 2011), with additional material still undescribed.

## Material and methods

Though a variety of sampling methods have been implemented in the Great Basin cave bioinventory, sciarids of the genus *Camptochaeta* were only obtained by hand collections, often using an aspirator. The sampling effort included entrance, twilight and dark zone sampling in 21 caves over 64 visits, and most collections are associated with data on light, temperature, and relative humidity, all collected with handheld meters, as well as data on subtrate, cave zone and elevation. Samples were preserved in the field in 70% ethanol, and later sorted and curated in the laboratory, with select material being distributed to appropriate taxonomic experts.

The material, kept in alcohol, was slide-mounted in “Euparal” after desiccation in absolute ethanol. The morphological terminology used and methods of measuring structures follow mainly Hippa and Vilkamaa (1991, 1994). The material is deposited in National Museum of Natural History, Washington, D.C (USNM), Illinois Natural History Survey Insect Collection, Champaign (INHS) and Zoological Museum, Finnish Museum of Natural History, Helsinki (MZH).

## Taxonomy

### Camptochaeta prolixa sp. n.

urn:lsid:zoobank.org:act:9289EB2A-6160-49E2-B2E4-69E67037868B

http://species-id.net/wiki/Camptochaeta_prolixa

#### Type locality.

USA, Nevada, White Pine County, Root Cave [39°00'N, 114°13'W], hand collection, 25.v.2006, S.J. Taylor, J.K. Krejca, M.E. Slay, G.M. Baker & B. Roberts.

#### Type material.

* Holotype male, *dissected, and mounted on microscope slide in Euparal. Original label: USA, Nevada, White Pine County, Root Cave, hand collection, 25.v.2006, S.J. Taylor, J.K. Krejca, M.E. Slay, G.M. Baker & B. Roberts (in USNM). *Paratypes*, dissected and mounted as the holotype: 1 male, same data but dry calcite floor, G.M. Baker, S.J. Taylor & J.K. Krejca (in USNM); 1 male, Nevada, White Pine County, Lehman Cave, 25.vi.2006, G.M. Baker, M.A. Horner & B. O'Doan (in INHS); 1 male, same locality but dry calcite wall, hand collection, 26.v.2006, S.J. Taylor, J.K. Krejca, M.E. Slay & G.M. Baker (in MZH); 2 males, Lehman Cave Annex, under rocks, hand collection, 25.v.2006, S.J. Taylor, J.K. Krejca, M.E. Slay B. Roberts, & M. Horner (in MZH); 1 male, Nevada, White Pine County, Cave 24, hand collection, 17.vii.2007, G.M. Baker & S.J. Taylor (in MZH). *Other material*. 1 female, Nevada, White Pine County, Lehman Cave, under rocks, hand collection, 25.v.2006, S.J. Taylor, J.K. Krejca, M.E. Slay, B. Roberts, & M. Horner (in MZH).

#### Description.

**Figure 1.**
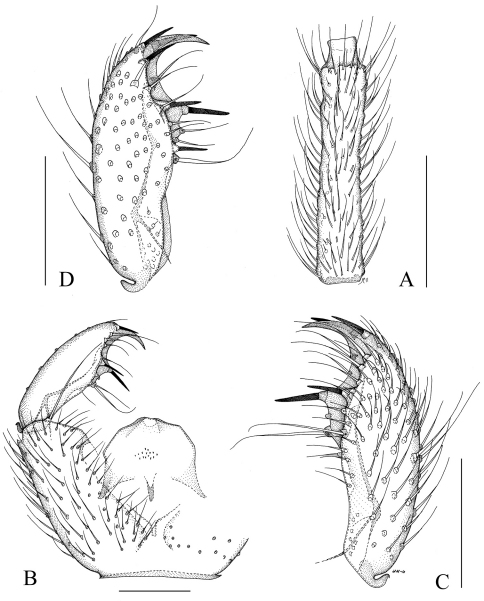
*Camptochaeta prolixa* sp. n. **A** Antennal flagellomere 4, lateral view **B** part of hypopygium, ventral view **C** and **D** Gonostylus, ventral view. **A–C** holotype, **D** paratype from Lehman Cave annex. Scale 0.1 mm.

 Male. **Head.** Brown, maxillary palpus very pale brown, antenna concolorous with face at base but paler towards apex. Eye bridge 1–2 facets wide, medially lacking ommatidia, sometimes narrowed into stripe. Face with 5–8 scattered setae. Clypeus with 1 seta. Maxillary palpus with 3 palpomeres; palpomere 3 longer than palpomere 1, palpomere 2 shortest; palpomere 1 with one (rarely 2) long sharp seta, with a dorsal pit with long sensilla; palpomere 2 with 3 (rarely 4) long sharp setae, 7–9 shorter truncate setae, palpomere 3 with 8–12 short truncate setae. Antenna long, antennal flagellomere 4, Fig. 1A, 4.0–5.8x as long as wide, the neck shorter than the width of flagellomere, the longest setae longer than the width of flagellomere. **Thorax.** Concolorous brown, setae pale. Anterior pronotum with 3–6 setae. Episternum 1 with 5–8 setae. **Wings.** Length 2.4–2.8 mm. Width/length 0.35–0.40. R1/R 0.85–1.05. c/w 0.65–0.70. r-m with 2–5 setae, bM non-setose. Halter pale brown. **Legs. **Yellowish, long. Metatarsus long, probasitarsomere as long as profemur; the modified vestiture of protibia pale, in patch in shallow depression. Protibial spur slightly longer than tibial width. **Abdomen. **Pale brown. Setae pale and long. **Hypopygium, **Fig. 1B–D. Brown, as abdomen. Gonocoxa slightly longer than gonostylus. The ventral setosity of gonocoxa sparse. Gonostylusnarrow, the mesial side impressed on apical third; the setosity sparse, apicomesially with a few elongated setae; with long apical tooth, with 2–3 megasetae in two groups, apically and subapically, megasetae straight, one of the subapical ones larger than others; mesially with 1–2 long flagellate setae. Tegmen simple, with indistinct subapical lateral shoulders, laterally slightly sclerotized. Aedeagal apodeme short, aedeagal teeth minute.

*Female*. Slightly larger than male, wing length 3.0 mm, without diagnostic characters.

#### Discussion.

* Camptochaeta prolixa *isvery similar to *Camptochaeta subcamptochaeta* found in central European caves, and the very similar *Camptochaeta pentacantha* found in the Altay, Russia. *Camptochaeta prolixa* differs from both by having a more slender gonostylus and by having the antennal flagellomeres longer, in most specimens more than five times as long as wide, with longer setosity; and by having a narrower eye bridge. *Camptochaeta prolixa *and resembles the European cave-dwelling *Camptochaeta ofenkaulis* by having long antenna and legs, but the gonostylus is remarkably different. Some specimens of C*. prolixa* show a tendency to a reduced eye bridge, which in addition to the long legs and antenna may be an accommodation to the subterranean mode of life.

Ten of the 11 specimens of *Camptochaeta prolixa* were collected in the dark zones of caves, and one in the twilight zone. Based on microhabitat-specific data for ten specimens: air temperature ranged from 6.6 to 13.0 C, average 10.9 C; relative humidity ranged from 82.6 to 92.4 %, average 87.7 %; light ranged from 0 to <1 lux, average 0.0 lux; and elevation ranged from 2089 to 2013 m, average 2228 m. All *Camptochaeta prolixa* specimens were collected in May (9 specimens) and July (2 specimens), even though sampling was carried out monthly in Lehman Caves. Most specimens were associated with bedrock or calcite walls or ceilings.

#### Etymology.

 The name is Latin (adjective), *prolixa*, streched out, referring to the very long extremities of the fly.

##### Records of *Camptochaeta* species in caves

*Camptochaeta mutua* (Johannsen, 1912)

9 males, USA, Arkansas, Stone County, Blanchard Springs Caverns, 27.iv.2002, M. Slay, G. Graening & K. Tinkle (in MZH).

*Camptochaeta mutua* was described from Ithaca, New York (Johannsen 1912) and is recorded from eastern USA and Canada (Newfoundland, Ontario, Quebec and also from Yukon (Hippa and Vilkamaa 1994).

*Camptochaeta pellax* Hippa & Vilkamaa, 1994

1 male, Nevada, White Pine County, Lehman Cave Annex, dry ceiling, hand collection, 25.v.2006, S.J. Taylor, J.K. Krejca, M.E. Slay B. Roberts, & M. Horner (in MZH); 1 male, White Pine County, Root Cave, calcite wall, hand collection, 25.v.2006, S.J. Taylor, G.M. Baker & B. Roberts (in INHS).

Two specimens of *Camptochaeta pellax* were collected from the dark zone of caves, with the following microhabitat-specific data: air temperature ranged from 10.9 to 13.0 C, average 11.95 C; relative humidity ranged from 84.4 to 88.6 %, average 86.5 %; light 0 lux; elevation ranged from 2089 to 2235 m, average 2162 m. *Camptochaeta pellax* is earlier known only from the type material from Colorado (Hippa and Vilkamaa 1994).

*Camptochaeta spicigera* Hippa & Vilkamaa, 1994

1 male, Nevada, White Pine County, Lincoln Mine, hand collection, 15.vii.2007, S.J. Taylor, J.K. Krejca, M.E. Slay, C.M. Slay (in MZH).

The Nevada specimen of *Camptochaeta spicigera* was collected in the entrance zone of a mine on wet rocks above water on the mine floor, with the following microhabitat-specific data: air temperature 9.7 C; relative humidity 52.5%; light 1755 lux; elevation 2621 m. *Camptochaeta spicigera* is earlier known only from the type material from Colorado (Hippa and Vilkamaa 1994).

## Discussion

The distributions of temperature and humidity data for the new species are consistent with the morphological evidence that this species is a troglobite. Only one specimen was found in the twilight zone, and with the exception of that specimen, the species was always associated with elevated relative humidity and stable temperatures consistent with deep-cave habitats.

Our description of a new *Camptochaeta* brings the number of recently described cave organisms from Great Basin National Park to five. The sampled caves span a range of 1724 to 3413 meters in elevation, crossing a variety of vegetation zones from to above timberline. Within the caves, there are a variety of microhabitats with varying levels of nutrient input and habitat stability. In addition, the Park is located in a relatively sparsely populated area, with few entomologists. A combination of these factors may account for the relatively high number of new species recently described from this area.

## Supplementary Material

XML Treatment for Camptochaeta prolixa
